# Community-Acquired *Klebsiella pneumoniae* Bacteremia: Global Differences in Clinical Patterns

**DOI:** 10.3201/eid0802.010025

**Published:** 2002-02

**Authors:** Wen-Chien Ko, David L. Paterson, Anthanasia J. Sagnimeni, Dennis S. Hansen, Anne Von Gottberg, Sunita Mohapatra, Jose Maria Casellas, Herman Goossens, Lutfiye Mulazimoglu, Gordon Trenholme, Keith P. Klugman, Joseph G. McCormack, Victor L. Yu

**Affiliations:** * National Cheng Kung University Medical College, Taiwan; † Veterans Administration Medical Center and University of Pittsburgh, Pittsburgh, Pennsylvania, USA; ‡ University of Queensland, Mater Adults Hospital, Brisbane, Australia; § Statens Serum Institut, Copenhagen, Denmark; ¶ South African Institute of Medical Research, Johannesburg, South Africa; # Rush-Presbyterian-St. Luke’s Medical Center, Chicago, Illinois, USA; ** Sanatorio San Lucas, Buenos Aires, Argentina; †† University Hospital, Antwerp, Belgium; ‡‡ Marmara University, Istanbul, Turkey

**Keywords:** Klebsiella, pneumonia, liver abscess, Taiwan, South Africa

## Abstract

We initiated a worldwide collaborative study, including 455 episodes of bacteremia, to elucidate the clinical patterns of *Klebsiella pneumoniae*. Historically, community-acquired pneumonia has been consistently associated with *K. pneumoniae*. Only four cases of community-acquired bacteremic *K. pneumoniae* pneumonia were seen in the 2-year study period in the United States, Argentina, Europe, or Australia; none were in alcoholics. In contrast, 53 cases of bacteremic *K. pneumoniae* pneumonia were observed in South Africa and Taiwan, where an association with alcoholism persisted (p=0.007). Twenty-five cases of a distinctive syndrome consisting of *K. pneumoniae* bacteremia in conjunction with community-acquired liver abscess, meningitis, or endophthalmitis were observed. A distinctive form of *K. pneumoniae* infection, often causing liver abscess, was identified, almost exclusively in Taiwan.

*Klebsiella pneumoniae* is among the most common gram-negative bacteria encountered by physicians worldwide. It is a common hospital-acquired pathogen, causing urinary tract infections, nosocomial pneumonia, and intraabdominal infections. *K. pneumoniae* is also a potential community-acquired pathogen. In this international collaborative study, we evaluated geographic differences and trends in three prominent presentations of community-acquired *Klebsiella* infection.

First, *K. pneumoniae* has been a recognized pulmonary pathogen since its discovery >100 years ago. The classic clinical presentation is dramatic: toxic presentation with sudden onset, high fever, and hemoptysis (currant jelly sputum). Chest radiographic abnormalities such as bulging interlobar fissure and cavitary abscesses are prominent. However, the incidence of community-acquired *Klebsiella* pneumonia has apparently declined in the United States (*1*,*2*). In studies from the 1920s to the 1960s, *K. pneumoniae* was considered an important cause of community-acquired pneumonia (*2*); however, in the last decade *K. pneumoniae* accounted for <1% of cases of pneumonia requiring hospitalization in North America (*3*,*4*).

Second, a striking clinical finding concerning a new manifestation of community-acquired *K. pneumoniae* infections has been documented. An unusual invasive presentation of *K. pneumoniae* infection, primary bacteremic liver abscess, has been described by numerous investigators in Asia; >900 patients with *Klebsiella* liver abscess have been reported from Taiwan in the last 10 years (*5*–*22*). In addition, case reports and small series from Korea, Singapore, Japan, India, and Thailand have been published (*23*–*32*). The Taiwanese patients with *K. pneumoniae* liver abscess have no history of hepatobiliary disease. Seventy percent of such patients have diabetes mellitus (*5*,*13*,*18*,*19*); 11% to 12% of the reported patients with *Klebsiella* liver abscess have other septic metastatic lesions, including pulmonary emboli or abscess, brain abscess, pyogenic meningitis, endophthalmitis, prostatic abscess, osteomyelitis, septic arthritis, or psoas abscess (*5*,*13*,*19*).

The third striking clinical observation is the preponderance of *K. pneumoniae* as a cause of community-acquired bacterial meningitis in adults in Taiwan, even in the absence of liver abscess or other sites of infection. The proportion of cases of culture-proven bacterial meningitis due to *K. pneumoniae* in one Taiwanese hospital increased from 8% during 1981 and 1986 to 18% during 1987 to 1995 (*33*). In contrast, in a recent large review only 3 (1.2%) of 253 cases of community-acquired bacterial meningitis from the Massachusetts General Hospital were due to *K. pneumoniae* (*34*).

Given these empiric observations, we established an international collaboration of researchers from each of the world’s populated continents. These investigators worked in large tertiary-care hospitals or hospitals serving veterans. One of our aims was to delineate in a single time period, with a consistent set of definitions, global differences in the clinical manifestations of serious *K. pneumoniae* infections. We also examined the influence of prior antibiotic use on these differences in *K. pneumoniae* infections.

## Methods

A prospective study of consecutive patients with community-acquired *K. pneumoniae* bacteremia was performed in 12 hospitals.^1^ The study period was January 1, 1996, to December 31, 1997. Records of patients >16 years of age with positive blood cultures for *K. pneumoniae* were reviewed, and a 188-item study form was completed. All items on the form were objective criteria, allowing standardization among medical centers. The study was observational in that administration of antimicrobial agents and other therapeutic management were controlled by the patient’s physician, not the investigators.

Community-acquired bacteremia was defined as a positive blood culture taken on or within 48 hours of admission. Severity of acute illness at the time of positive blood cultures was assessed by a previously validated scoring system, based on mental status, vital signs, need for mechanical ventilation, and recent cardiac arrest (Pitt bacteremia score) (*35*). Type of infection was defined as pneumonia, urinary tract infection, meningitis, incisional wound infection, other soft tissue infection, intraabdominal infection, and primary bloodstream infection, according to Centers for Disease Control and Prevention definitions (*36*). In addition, distinctive sites of *K. pneumoniae* bacteremia were further defined as liver abscess, meningitis, or endophthalmitis. Liver abscesses were defined by the coexistence of blood cultures positive for *K. pneumoniae* and evidence of an intrahepatic abscess cavity by ultrasonography or computed tomography. Meningitis was defined as culture of *K. pneumoniae* from cerebrospinal fluid, and endophthalmitis was defined as decreased visual acuity, pain, hypyon, or severe anterior uveitis in a patient concurrently bacteremic with *K. pneumoniae*. Death was defined as including deaths from all causes within 14 days of the date the first positive blood culture for *K. pneumoniae* was obtained.

Blood cultures of *K. pneumoniae* were sent by participating hospitals on nutrient agar slants to a central study laboratory in Pittsburgh, where the identity of each isolate as *K. pneumoniae* was confirmed by the Vitek GNI system (Biomerieux Vitek, Hazelwood, MO). Extended-spectrum beta-lactamase (ESBL) production was defined phenotypically by broth dilution as a ≥3 twofold concentration decrease in MIC for either cefotaxime or ceftazidime tested in combination with clavulanic acid compared with the MIC when tested alone. The protocol was reviewed and approved by Institutional Review Boards according to local requirements.

All data were entered into a central database (PROPHET version 5.1; BBN Systems and Technologies Corporation, Cambridge, MA). Contingency data were analyzed by two-tailed chi-square or Fisher’s exact tests, and continuous data were analyzed by Student *t* test or Mann-Whitney U test.

##  Results

Two hundred two (44.4%) of 455 episodes of *K. pneumoniae* bacteremia during the study period were community-acquired cases. The percentage of cases of *K. pneumoniae* bacteremia that were community acquired in each study country differed strikingly: 96 (68%) of 142 in Taiwan, 25 (43%) of 68 in the United States, 28 (39%) of 71 in Australia, 40 (34%) of 116 in South Africa, 6 (22%) of 27 in Europe, and 7 (17%) of 41 in Argentina. *K. pneumoniae* bacteremia in Taiwan was significantly more likely to be community acquired than was bacteremia in the other countries combined (68% vs. 36%, p=0.0001).

The characteristics of patients with community-acquired *K. pneumoniae* bacteremia from Taiwan, South Africa, and the rest of the world were compared (Table 1). The source of bacteremia in community-acquired cases was geographically distinctive (Table 2). Pneumonia was the most common infection worldwide, accounting for 57 (28%) of 202 cases. However, 53 (93%) of 57 of all cases of community-acquired *K. pneumoniae* pneumonia occurred in Taiwan and South Africa.

**Table 1:** Clinical characteristics of patients with community-acquired *Klebsiella pneumoniae* bacteremia from Taiwan, South Africa, and other countries

Clinical characteristics	Taiwan (n=96)	South Africa (n=40)	Other countries (n=66)	p value^a^
Age (mean, years)	58.8	47.2	59.9	0.014
Female, n (%)	35 (38%)	17 (42%)	24 (36%)	NS
Underlying diseases, n (%)				
Diabetes mellitus	38 (40%)	8 (20%)	19 (29%)	0.06
Liver disease	33 (34%)	6 (15%)	12 (18%)	0.02
Alcoholism	12 (12%)	3 (8%)	1 (2%)	0.08
Malignancy	15 (16%)	3 (8%)	25 (38%)	0.0002
HIV infection	0 (0%)	7 (18%)	0 (0%)	0.0001
Chronic renal failure	6 (6%)	2 (5%)	6 (9%)	NS
Organ transplant	0 (0%)	0 (0%)	7 (11%)	0.0006
Corticosteroid use	5 (5%)	1 (3%)	7 (11%)	NS
No underlying disease	22 (23%)	21 (52%)	24 (36%)	0.003
Critically ill^b^	30 (31%)	12 (30%)	2 (3%)	0
Death rate at 14 days, n (%)	30 (31%)	24 (60%)	8 (12%)	0

^a^p values refer to differences between the three regions; NS = not significant at p >0.20.

^b^Critically ill defined as Pitt bacteremia score >4.

**Table 2:** Worldwide differences in the sites of infection associated with community-acquired *Klebsiella pneumoniae* bacteremia

Infection site	Taiwan^a^ (n=96)	South Africa (n=40)	Other countries (n=66)	p value^b^
Pneumonia	28 (29%)	25 (62%)	4 (6%)	0
Liver abscess	17 (18%)	0	1 (2%)	0
Endophthalmitis	1 (1%)	0	0	NS
Meningitis	5 (5%)	2 (5%)	0	0.06
Urinary tract infections	14 (15%)	4 (10%)	25 (38%)	0.0003
Acute cholangitis	13 (14%)	0	12 (18%)	0.02
Intravascular catheter-related infections	0	2 (5%)	11 (17%)	0
Skin and soft tissue infections	5 (5%)	1 (3%)	4 (6%)	NS
Spontaneous bacterial peritonitis	7 (7%)	0	1 (2%)	0.06
Intraabdominal abscess	2 (2%)	2 (5%)	2 (3%)	NS
Other	1 (1%)	0	2 (3%)	NS
No primary site evident	7 (7%)	5 (12%)	4 (6%)	NS

^a^Four Taiwanese patients had more than one site of infection: pneumonia and liver abscess (2), liver abscess and meningitis (1) and pneumonia and endophthalmitis (1). One South African patient had both pneumonia and meningitis.

^b^p values refer to the differences between the three regions; NS = not significant at p >0.20.

Antibiotics had been used for >24 hours before admission in 21 (10%) of 202 patients. Prior antibiotic use was significantly lower in Taiwan (4 [4%] of 96 patients) and South Africa (2 [5%] of 40) than in the other countries (15 [23%] of 66) (p=0.0003). In four patients, the prior antibiotic was an oxyimino-containing cephalosporin (two in South Africa, one in Argentina, and one in Taiwan).

ESBL production was detected in 7 (3.5%) of 202 community-acquired strains compared with 78 (30.8%) of 253 hospital-acquired strains (p<0.00001). None of the patients with community-acquired ESBL-producing strains had recently received an oxyimino-containing cephalosporin. However, of the seven patients with community-acquired ESBL-producing *K. pneumoniae* bacteremia, only one (a patient with pneumonia from Africa) had no recent hospital exposure. The countries from which community-acquired ESBL-producing *K. pneumoniae* isolates were collected included Africa (three isolates), Turkey (two), the United States (one), and Australia (one). Twenty-five bloodstream isolates were ciprofloxacin resistant, of which 7 (28%) of 25 were community acquired. All these seven patients had serious underlying disease and frequent hospitalizations or nursing home admissions, and none had received a quinolone in the 14 days before hospital admission. Five of seven patients with community-acquired, ciprofloxacin-resistant *K. pneumoniae* bacteremia were from Taiwan.

### Community-Acquired Pneumonia

Community-acquired pneumonia due to *K. pneumoniae* was significantly associated with alcoholism (p=0.007); 18% of patients with pneumonia were alcoholics as defined by their physicians, compared with 4% with other sources of *K. pneumoniae* bacteremia (Table 3). However, no patient with community-acquired *K. pneumoniae* bacteremic pneumonia outside South Africa or Taiwan was an alcoholic; of these patients, one was neutropenic, two were nursing home residents with neurologic impairment (ages 81 and 90), and one was a Vietnamese immigrant to Australia with no underlying illness.

**Table 3:** Comparison of the characteristics of patients with community-acquired bacteremic pneumonia due to *Klebsiella pneumoniae* and other patients with community-acquired *K. pneumoniae* bacteremia: association between pneumonia and alcoholism and residence in South Africa

Characteristic	Bacteremic pneumonia (n=57)	Bacteremia without pneumonia (n=145)	p value^a^
Resides in South Africa	25 (44%)	15 (10%)	<0.001
Age (years)	53.6	58.6	0.07
Serum creatinine^b^ (mg/dL)	2.1	2.3	0.2
Blood urea nitrogen^b^ (mg/dL)	34.8	37.9	NS
Liver function tests^b,c^			
Serum albumin (g/mL)	2.8	3.1	0.05
Serum bilirubin (mg/dL)	2.8	2.9	NS
AST (IU/mL)	174	303	NS
ALT (IU/mL)	115	189	NS
Underlying disease			
Diabetes mellitus (%)	12 (21%)	53 (37%)	0.03
Alcoholism (%)	10 (18%)	6 (4%)	0.007
Malignancy (%)	7 (12%)	36 (25%)	0.05
HIV infection (%)	6 (10%)	1 (1%)	0.002
No underlying disease	23 (40%)	44 (30%)	NS (0.17)
Critically ill	21 (37%)	23 (16%)	0.001
Death rate at 14 days (%)	31 (54%)	32 (22%)	0.0001

^a^NS = not significant at p >0.20; AST = aspartate aminotransferase; ALT = alanine aminotransferase.

^b^Laboratory values are those taken on first visit to a health-care provider; for continuous variables, the figures in the table are mean values.

^c^Bacteremic patients with liver cirrhosis, acute cholangitis, and liver abscess were excluded from the analysis of liver function tests.

Community-acquired pneumonia due to *K. pneumoniae* was significantly associated with HIV infection on univariate evaluation (p=0.002). Of the seven patients with HIV infection and *K. pneumoniae* bacteremia (all from Africa), six had community-acquired pneumonia. Community-acquired pneumonia due to *K. pneumoniae* was not associated with underlying liver disease, chronic renal failure, receipt of chemotherapy for malignant disease, or receipt of corticosteroids.

Multivariate analysis showed that residing in Africa (p=0.0001) or Taiwan (p=0.0046) and being an alcoholic (p=0.04) were significantly associated with community-acquired *K. pneumoniae* pneumonia. HIV infection was not independently associated with pneumonia (p=0.23). The death rates from community-acquired pneumonia due to *K. pneumoniae* were 54% in Taiwan and 56% in South Africa.

### A Distinctive *K. pneumoniae* Bacteremia Syndrome

Twenty-five patients had a distinctive syndrome of *K. pneumoniae* bacteremia, which was defined by the presence of *K. pneumoniae* bacteremia in conjunction with liver abscess, endophthalmitis, or meningitis. Of these patients, 88% (16 with liver abscess, 4 with meningitis, 1 with liver abscess and meningitis, and 1 with endophthalmitis) were from Taiwan, compared with 12% from the other countries combined (2 with meningitis from South Africa and 1 with liver abscess from Belgium) (p=0.0001).

Twelve (67%) of 18 patients with liver abscess had diabetes mellitus. On univariate analysis, residing in Taiwan (p=0.0001) and having diabetes mellitus (p=0.001) were significantly associated with community-acquired *K. pneumoniae* liver abscess. Patients with liver abscess were more likely to have renal failure, but this association was not statistically significant (p=0.09). There was no association between liver abscess and gender, age, previous antibiotic use, or presence of underlying liver disease. Multivariate analysis showed that residence in Taiwan (p=0.0034), diabetes mellitus (p=0.0058), and renal failure (p=0.0178) were significantly associated with the presence of liver abscess.

Patients with any of the distinctive manifestations of *K. pneumoniae* (liver abscess, meningitis, or endophthalmitis) were compared with patients with other community-acquired infections (Table 4). These complications were significantly associated with diabetes mellitus (60% vs. 28%, p=0.0015) and living in Taiwan (88% vs. 42%, p=0.0001).

**Table 4:** Comparison of the characteristics of patients with distinctive infections associated with community-acquired *Klebsiella pneumoniae* bacteremia (liver abscess, meningitis, and endophthalmitis) and other patients with community-acquired *K. pneumoniae* bacteremia: association with diabetes mellitus and residence in Taiwan

Characteristic	Liver abscess, endophthalmitis or meningitis (n=25)	Other community-acquired bacteremia (n=177)	p-value^a^
Resides in Taiwan	22 (88%)	74 (42%)	0.0001
Age (years)	55.5	57.4	NS
Serum creatinine^b^ (mg/dL)	2.2	2.2	NS
Blood urea nitrogen* (mg/dL)	35.7	37.2	NS
Underlying disease			
Diabetes mellitus (%)	15 (60%)	50 (28%)	0.0015
Chronic renal failure (%)	3 (12%)	11 (6%)	NS
Underlying liver disease (%)	5 (20%)	46 (26%)	NS
Chronic hepatitis B virus infection	0	9 (5%)	NS
Hepatitis C virus infection (%)	1 (4%)	8 (5%)	NS
Alcoholism (%)	2 (8%)	14 (8%)	NS
Malignancy (%)	0	43 (24%)	0.006
No underlying disease	6 (24%)	61 (34%)	NS
Critically ill^c^	9 (36%)	35 (20%)	0.07
Death rate at 14 days (%)	8 (32%)	54 (31%)	NS

^a^NS = p >0.20.

^b^Laboratory values are those taken on first visit to a health-care provider; for continuous variables, the figures in the table are mean values.

^c^Critically ill defined as Pitt bacteremia score ≥4.

## Discussion

When pneumonia due to *Klebsiella* was first described by Friedlander in 1882, he believed it to be the most common cause of bacterial pneumonia (*37*). Although this concept was soon refuted in favor of pneumococcus, from the 1930s through the 1960s, 10 to 50 cases of *Klebsiella* pneumonia were reported each year by large hospitals in the United States (*2*). U.S. textbooks of medicine continue to list *K. pneumoniae* as an important cause of community-acquired pneumonia (*38*,*39*).

In our prospective study, we found only four cases of community-acquired *K. pneumoniae* pneumonia in 2 years in nine large hospitals from the United States, Australia, Europe, and Argentina. The hospitals surveyed included an inner-city veterans hospital in the United States and two large inner-city public hospitals in Australia. These three centers care for large numbers of indigent and alcoholic patients.

Recently published reports from the United States, Israel, and Europe support our observations. Neither Vergis et al. (*40*) from the United States nor Lieberman et al. (*41*) from Israel found a single case of *K. pneumoniae* pneumonia in large multicenter studies of community-acquired pneumonia in the 1990s. Nine European studies published since 1990 show that only 14 (2.3%) of 621 patients admitted with severe community-acquired pneumonia requiring intensive-care unit admission had *K. pneumoniae* as the presumptive etiologic agent (*42*–*50*). In contrast, *K. pneumoniae* continues to be associated with community-acquired pneumonia in Africa and Asia. In our study, we observed 28 cases in Taiwan and 25 in South Africa, accounting for 29% and 62% of all cases of community-acquired *K. pneumoniae* bacteremia in South Africa and Taiwan, respectively (Table 2). Recent studies from Taiwan, Singapore, and South Africa corroborate these findings. In a Taiwanese study, *K. pneumoniae* accounted for 34% of 41 cases of community-acquired bacteremic pneumonia (*51*). *K. pneumoniae* was the cause of 15% of community-acquired pneumonia requiring intensive-care unit admission in Singapore (*52*). *K. pneumoniae* was found to be the cause of pneumonia in 32% of African patients with severe community-acquired pneumonia requiring intensive-care unit admission in Johannesburg (*53*) and 11% of patients requiring intensive-care unit admission in Cape Town (*54*).

In our study, 18% of patients with community-acquired *K. pneumoniae* pneumonia were alcoholics (p=0.007) (Table 3). Alcoholics in Africa and Asia may have limited access to health care (perhaps including reduced access to antibiotics) compared with those in the Americas, Europe, and Australia, and may have respiratory symptoms later. A weakness of our study is that we were not able to ascertain the duration of symptoms before each patient was hospitalized. However, a recent study of aborigines from rural northern Australia (35% of whom were alcoholics and most of whom had suboptimal access to health-care facilities) showed that none of 90 admitted to hospital with community-acquired pneumonia had *K. pneumoniae* infection (*55*). The hypothesis that *Klebsiella* pneumonia is related to poor primary health care for alcoholics may therefore be less plausible.

Bacteremic *K. pneumoniae* liver abscess occurred almost exclusively in patients from Taiwan (Table 2), consistent with a growing number of reports from Asia describing this distinctive type of infection. *K. pneumoniae* was the most common cause of liver abscesses in Taiwan, Singapore, and Korea in reports from 1990 to 1999 (*5*,*9*,*24*,*31*,*32*); similarly, numerous reports of liver abscess have recently been published from Hong Kong, Thailand, and Japan (*23*,*25*–*30*). In total, >900 patients with *K. pneumoniae* liver abscess have been reported from Asian countries in the last 10 years; in contrast, reports of only 23 patients with this condition have been published from regions outside Asia in this same period (*56*–*63*).

*K. pneumoniae* meningitis in adults has also been infrequently reported from North America, Europe, and Australia, in contrast to Taiwan. In our study, five cases of bacteremic *K. pneumoniae* meningitis were in Taiwanese patients and two in African patients (Table 2). Four (57%) of 7 patients with meningitis had prior diabetes mellitus. Meningitis caused by *K. pneumoniae* in the United States, Australia, and Europe is most often hospital acquired and associated with prior neurosurgical procedures or instrumentation. However, of 115 cases of *K. pneumoniae* meningitis reported from Taiwan (*33*,*64*,*65*), 84% were community acquired, and 64% of cases had concurrent *Klebsiella* bacteremia. Unlike pyogenic liver abscess, the clinical course was fulminant, with a death rate of 57% (*33*,*64*,*65*). The death rate from bacteremic *K. pneumoniae* meningitis in our series was 71%.

We found only one patient (an alcoholic from Taiwan) with *K. pneumoniae* bacteremia and endophthalmitis. *K. pneumoniae* endophthalmitis is also likely to be far more common in Asia than elsewhere; >50 cases have been reported in the last 10 years from Asia (*6*,*10*,*11*,*19*,*27*,*66*–*70*) compared with only 10 from the United States, Europe, and Australia (*57*,*60*,*62*,*71*–*76*). More than 50% of previously reported Asian patients with *K. pneumoniae* endophthalmitis have had concurrent liver abscess (*6*,*10*,*11*,*19*,*27*,*66*–*70*).

The reason for the geographic preponderance of these severe manifestations of *K. pneumoniae* infections in Asia is unknown. The geographic diversity of *Klebsiella* infections possibly results from interaction between bacterial variables, host variables (for example, defects in host defense caused by diabetes mellitus or alcoholism), socioeconomic factors, and possibly genetic susceptibility in different racial groups. We are studying the phenotypic and genotypic differences in *K. pneumoniae* causing different disease manifestations in different countries. Because no more than three hospitals from each country were included in our study, our results may not necessarily be generalizable to hospitals in other regions. In addition, other countries in the same continent (e.g., other countries in Asia or eastern Europe) were not studied but may have different clinical patterns compared with the study country.

In summary, our results challenge the classic view of serious *Klebsiella* infections. In the United States, Europe, Argentina, and Australia, we have observed that hospital-acquired *K. pneumoniae* infections predominate, with community-acquired bacteremia being caused by urinary tract infection, vascular catheter infection, and cholangitis. Classic community-acquired pneumonia is no longer an important entity in these regions. In South Africa, pneumonia (especially in alcoholics) continues to be an important community-acquired infection. In Taiwan, community-acquired pneumonia persists, and distinctive infections such as liver abscess, endophthalmitis, and meningitis have emerged as substantial public health problems.

## Footnotes

1 United States: Pittsburgh Veterans Affairs Medical Center, Pittsburgh, Pennsylvania, and Rush-Presbyterian-St. Luke’s Medical Center, Chicago, Illinois; Taiwan: National Cheng Kung University Medical College, Tainan; Australia: Royal Brisbane Hospital, Mater Adults Hospital, and Greenslopes Private Hospital, all in Brisbane; South Africa: Hillbrow Hospital and Baragwanath Hospital, both in Johannesburg; Turkey: Marmara University Hospital, Istanbul; Belgium: University Hospital, Antwerp; and Argentina: San Lucas Hospital and Comunidad Olivos Hospital, both in Buenos Aires.
